# Microscopic characterization of level 3 surgical masks in a simulation model of dental care: a preliminary study

**DOI:** 10.1186/s12903-025-07077-w

**Published:** 2025-10-21

**Authors:** Maíra Prado, Leonardo Pereira Pacheco, Marina Carvalho Prado, João Victor Frazão Câmara, Silvia Renata de Souza Marski, Juliana do Nascimento Lunz, Braulio Archanjo, Carlos Alberto Achete, Renata Antoun Simão

**Affiliations:** 1 Postgraduate Program in Dentistry, Veiga de Almeida University (UVA), Rio de Janeiro, RJ Brazil; 2 Department of Materials Science and Engineering, Federal University of Rio de Janeiro (COPPE UFRJ), Rio de Janeiro, RJ Brazil; 3 Saarland University, Homburg Saar, Germany; 4 Materials Metrology Division (DIMAT), National Institute of Metrology Quality and Technology (INMETRO), Duque de Caxias, RJ Brazil

**Keywords:** Aerosols, Cross infection, Dental high-speed equipment, Microscopy, Respiratory protective devices

## Abstract

This study evaluated the morphology of level 3 surgical masks before and after aerosols’ passage using a simulation model of dental care with human breathing system to evaluate the aerosol filtering efficacy, as close as possible to the reality of a dental clinical setting. Methods: Eight groups of level 3 surgical masks were selected. The model used for analysis consisted of a human head prototype, a respirator system (artificial trachea, pneumatic plunger, and spirometry syringe), and a dummy head with artificial dental arch. Masks were exposed to 5 min of aerosol generation and the number of specimens with aerosol passage and total areas of aerosol pigmentation were evaluated. The microscopic analysis of masks using optical microscopy, helium ion microscopy and scanning electron microscope were performed before and after aerosols’ passage. Results: All groups presented aerosols passage (55–88%). The areas of aerosol passage revealed differences between groups (*p* < 0.05). In microscopy analysis, the inner and outer layers of masks showed a spunbond processing technique; the middle layer differed among groups of masks showing spunbond or meltblown processes, confirming what was reported by manufactures. Conclusion: The inner and outer layers of masks are morphologically similar, different to the middle layer. All level 3 surgical masks presented high levels of aerosols passage. Clinical significance: This study highlights the need for more effective surgical masks in dental care settings. Understanding the morphological differences between mask layers can inform improvements in mask design for better protection for healthcare professionals.

## Introduction

During dental care, the high-speed handpiece is routinely used for tooth drilling. The overheating of teeth and periodontal tissues are controlled by water and air [1, 2]. However, the specific form of oral cavity and the force of cooling water spray dispersedly ejects aerosols from the patient’s mouth over the dental clinical setting [3, 4]. The produced aerosols are generally infected (~ 75%) and may contain saliva, tooth fragments, dental plaque, blood and oral microorganisms [5, 6]. The oral cavity is a unique environment that normally has a wide diversity and a high concentration of dangerous pathogens [7] and the dental drilling produces approximately ~ 95% of particles capable to achieve the respiratory tract of the dentist [5, 6, 8]. Therefore, the dentist is at high-risk of cross-contaminations, especially in high-risk indoor settings [3, 9]. As a result, an increase in viral and bacterial infections was reported among dentists since the advent of the high-speed handpiece [3, 10, 11]. Protective masks have been endorsed as an integral part of precautions for dental care procedures and level 3 surgical masks (SM) have been indicated as the American Society for Testing & Materials (ASTM) standard protocol for dentists [9]. 

Dentists are daily exposed to different infectious microorganisms, and this risk was highlighted with the extremely contagious severe acute respiratory syndrome coronavirus 2 (SARS-CoV-2) [4]. With the discovery of the virus, after the lockdown period, dentists returned their professional activities and the recommendation was to use a combination of N95 respirators, a level 3 SM, and a face shield [9, 12, 13]. However, over time, with the vaccination and the population becoming immunized, dentists abandoned the use of respirators and face shields and returned to using only level 3 SM. Level 3 masks are designed for mechanical filtration of pathogens during procedures with moderate or heavy amounts of fluid spray, blood, or aerosol exposure (ASTM F2100). Nevertheless, the filtering efficiency of these masks ranges widely in the literature [14]. – [15].

Over the years, several methodologies have been used to evaluate dental aerosols and spatter, including the use of dyes [16–18], and bacterial contamination [19–21]. Although several studies have already been conducted in the literature, this topic gained greater relevance and visibility with the Coronavirus (COVID-19) pandemic. In 2020, Allison et al. proposed a new methodology, using fluoresceine sodium salt as dyer, to evaluate dental aerosols and spatter produced during different clinical procedures in filter papers [22]. The following year, in 2021, Llandro et al. evaluated the presence of splatter and aerosols contamination in filter papers during orthodontic debonding [23]. Regarding SM, Gund et al. observed microbiological contamination of masks during dental procedure and the transfer of microorganisms from the mask to the hands [24].

The real performance of level 3 SM during dental care is a considerably important matter for the development of more efficient strategies against cross-infections of dentists. There is a lack of previous in vitro studies evaluating conditions such as the distance and position of professionals, associated with their respiratory and the airflow rates, and the specificity of aerosol-generating procedures. These are relevant aspects impacting the analysis of filtering properties of masks. More sophisticated measurement methods for in vitro studies and up-to-date studies using current dental equipment are clearly needed. It is critical to do so mainly given the recent pandemic coupled with the asymptomatic disease carriers who are highly likely to undergo dental treatments [7]. Additionally, no studies were found evaluating the morphology of level 3 SM, which are commonly used in a dental environment, under simulated clinical conditions using a system to reproduce breathing and its impact. Therefore, the aim of this study was to evaluate the morphology of level 3 SM before and after aerosols’ passage using a simulation model of dental care with human breathing system to evaluate the aerosol filtering efficacy, as close as possible to the reality of a dental clinical setting.

## Materials and methods

### Groups of SM

Eight commercial brands of level 3 SM were selected for analysis: MaxClean (Biodont, Maringá, PR, Brazil), Descarpack (Descarpack Disposables, Centro Ilhota, SC, Brazil), Elite PROFESSIONAL (Elite Professional; CMPC Improvements, Caieiras, SP, Brazil), Sol-Millennum (Sol-Millennum Brazil, Ribeirão Preto, SP, Brazil), Neve (Neve Premium, Bragança Paulista, SP, Brazil), Ortho Pauher (Ortho Pauher, Recife, PE, Brazil), Phitta mask (Phitta, Centro Rio dos Cedros, SC, Brazil), and Fava Mask (Fava Mask; Fava Indústria, São Paulo, SP, Brazil). The analyzes were performed on 3 batches of each brand, in triplicate (*n* = 9).

### Development of simulation model of dental care environment

**Fig. 1:**
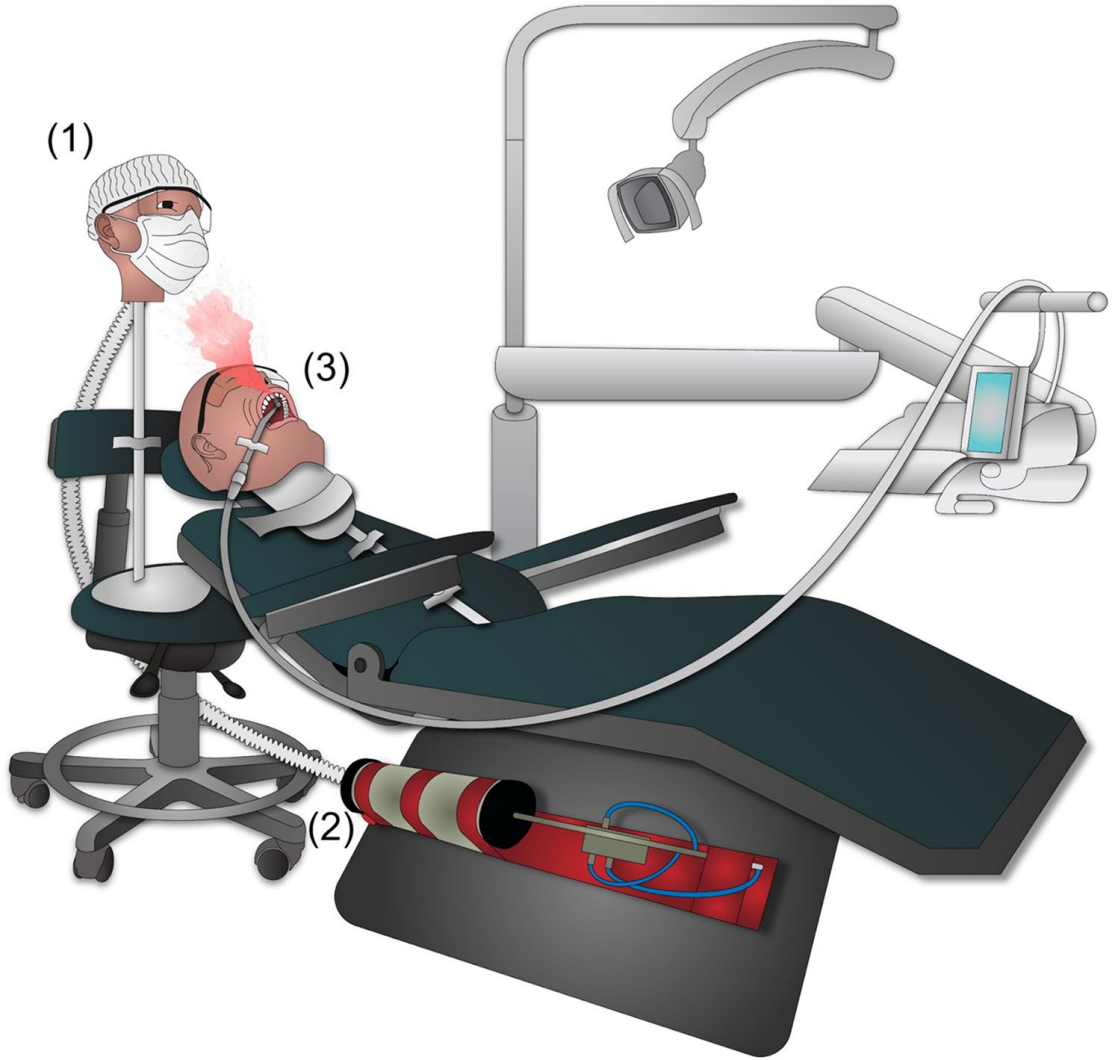
Representative figure of simulation model of dental care environment with breathing system. The system mainly consisted of: (1) a human head prototype used to attach the surgical masks evaluated, simulating the dentist, (2) an artificial respirator composed of a pneumatic plunger and a spirometry syringe, (3) a dummy head used in dentistry classes for laboratory simulation of dental care, which was associated to an artificial dental arch

The simulation model designed for analysis consisted of (Fig. 1): (1) a human head prototype used to attach the SM evaluated, simulating the dentist, (2) a respirator system composed of an artificial trachea, a pneumatic plunger, and a spirometry syringe, (3) a dummy head used for laboratory simulations of dental care, which was associated to an artificial dental arch, and (4) tubes and connections.

### Production of the human head prototype to simulate the dentist

**Fig. 2:**
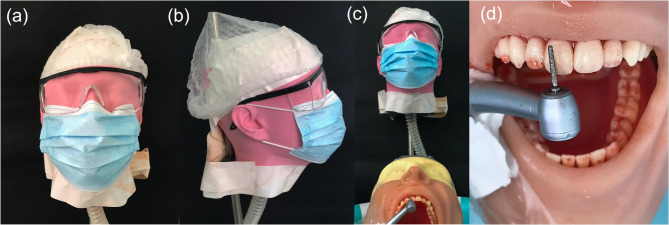
Simulation model of dental care environment. **a** Frontal and **b** lateral photos of printed human head prototype simulating the dentist. **c** Dental care position of the dentist based on anthropometric analysis and ergonomics principles. **d** Simulation of dental crown preparation of a maxillary central incisor using a high-speed handpiece

The human head prototype was produced using polymer acrylonitrile butadiene styrene (ABS) through the Fused Deposition Modeling/Fused Filament Fabrication (FDM/FFF) 3D printing technique (Funmat HT 3D Printer; Intamsys Technology CO. LTD., Shanghai, China). The prototype was produced in anatomical proportions similar to those of a human (Fig. 2a-b).

### Development of breathing system of the dentist

The breathing simulator consisted of a pneumatic plunger coupled to a 3 L calibrated spirometry syringe (Proarlife, Santo André, SP, Brazil). An adult silicone artificial trachea (22 × 120 cm; Prevtech, São Paulo, São Paulo, Brazil) was connected to both the spirometry syringe and the dentist through a hole on the posterior part of the human head prototype. The artificial trachea was placed exiting the mouth of the dentist, thus allowing the ventilation simulation. The plunger’s range of mobility and its precession frequency controlled the simulated tidal volume (V_T_) and the simulated respiratory rate (RR), respectively. Thereby, the length of the movement made by the plunger allowed a precise control of V_T_ in the spirometry syringe. Inspiratory and expiratory volumes were set with compatible physiological values, as well as RR [12–20 respirations per minute (rpm)] [25]. 

### Simulation of dentist during dental care

To obtain a reliable simulation of dental care environment and to standardize testing, an anthropometric analysis of dental professionals was previously accomplished as follow: the height of the human head prototype simulating the dentist, set at 1.67 m, was calculated considering the average of 142 undergraduate students at a Dental Medicine School (35 males and 107 females). Next, the average of sitting position height of the dentist on the chair was estimated in 57 cm by measuring 4 students with the determined height, 1.67 m, in a 12-hour working position and respecting the principles of ergonomics (Fig. 2c).

### Simulation of dental procedure

For visualization of aerosols, the water of the dental equipment was dyed with a red artificial dye (Tempemar Comercial de Alimentos LTDA, Três Rios, RJ, Brazil) that was soluble in water at a ratio of 10 ml/l [26]. Next, a piece of white bond paper measuring 8.5 × 8.5 cm^2^ was centrally fixed on the inner part of each mask, under the nose clip, aiming the visualization of the areas pigmented by the dye to characterize the aerosols’ passage. All SM were attached to the mouth of the dentist head prototype at a time and exposed to uninterrupted 5 min of aerosol generation. Aerosols were generated by simulating the preparation of a dental crown in a maxillary central incisor presented in the artificial dental arch of the dental dummy head (Pronew Top Dentística, São Gonçalo, RJ, Brazil) used to simulate the patient (Fig. 2d). The dental dummy was fixed to a dental chair (Versa Max Plux; Dabi Atlante, Ribeirão Preto, SP, Brazil), positioned 70 cm from the ground. A high-speed dental handpiece (3NS-kavo academic kit; KaVo Dental, Biberach an der Riss, Germany) was used with a >30 ml/min water flow for optimal cooling of a 4138-diamond drill (KG Sorensen, Cotia, SP, Brazil) [26]. The distance between the dentist and the the patient’s head was set at 10 cm. High suction was not used. The experiment was carried out in a closed room, with a temperature of 23 °C.

### Qualitative analysis of masks filtering

The presence or absence of red staining color on the piece of paper placed within each specimen indicated the passage of aerosols through masks.

### Quantitative analysis of masks filtering

**Fig. 3:**
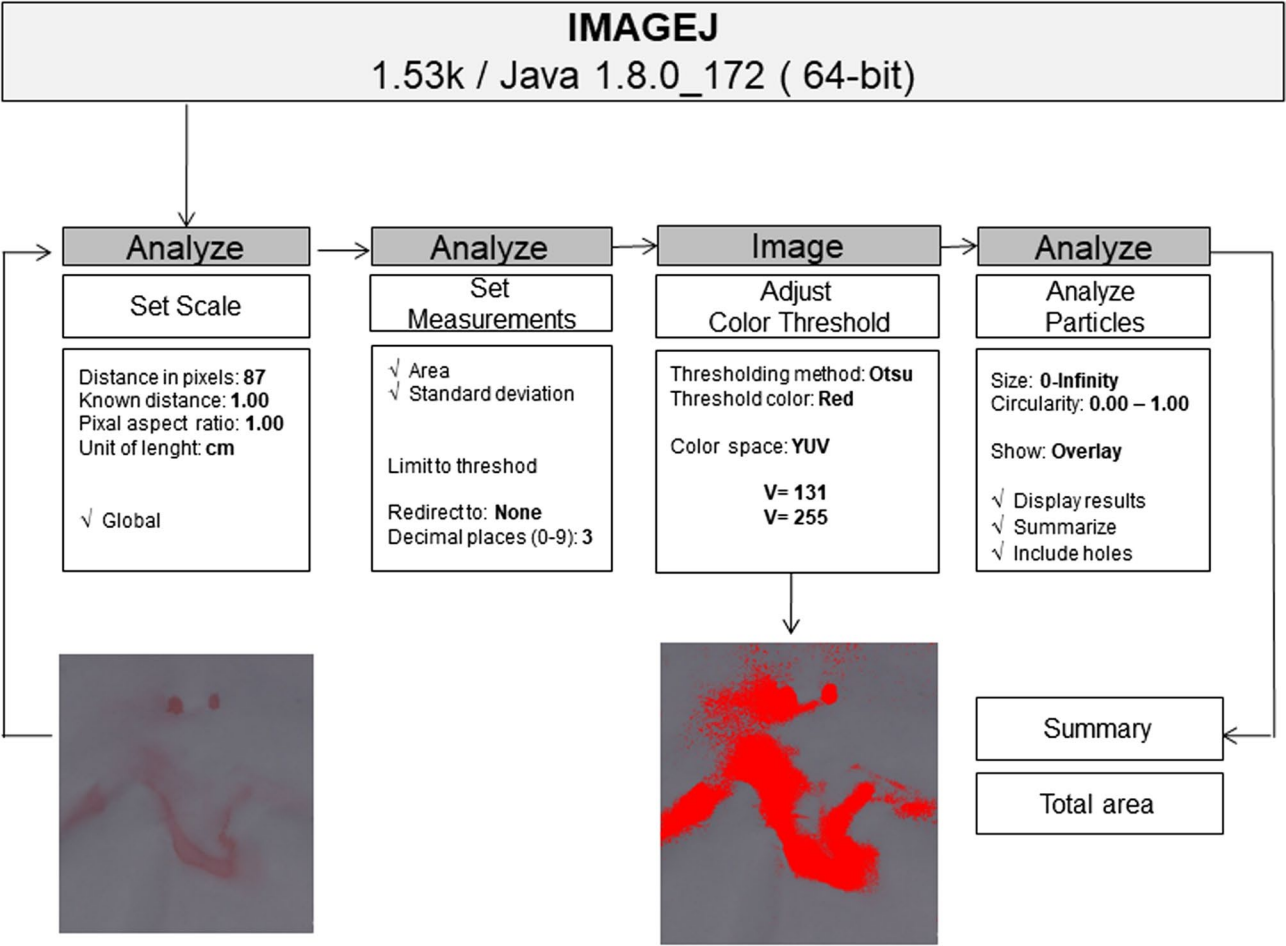
Image processing stages for quantitative analysis of aerosols passage through masks in ImageJ software v. 1.53 k (Wayne Rasband, National Institutes of Health, USA)

The surface of all papers was photographed (Canon EOS Rebel T4i; Canon São Paulo, SP, Brazil) with 100 mm macro lens (Canon Macro Ring Lite MR 14 EX, São Paulo, SP, Brazil), f/2.2 aperture, 1/200 second shutter speed, ISO 200 and 1/8 manual flash. Then, the pigmentation area on each paper was evaluated using ImageJ v 1.53 k (Wayne Rasband, National Institutes of Health, USA) (Fig. 3). The results were analyzed by non-parametric tests, Kruskal-Wallis and Dunn. A significance level of 5% was adopted.

### Microscopy analysis

Optical microscope (Stereo Microscopes Stemi 305/508; Carls Zeiss), helium ion beam microscope [helium ion microscopy (HIM) – Zeiss Orion Nanofab] and scanning electron microscope (SEM, Nova NanoLab™ DualBeam FIB) were used for morphological characterization. SM were evaluated before and after aerosols filtration test. For optical microscopy (40x) and HIM, the layers of each mask were separated into inner, middle, and outer layers and analyzed separately. For HIM, a thin layer of amorphous carbon (20 nm) was deposited on the samples to increase the conductivity. The images were acquired with two different field of views, 200 and 950 microns.

Cross-section images were done with SEM. Samples were immersed in liquid nitrogen for few minutes to obtain a flatter section using a scissor. Prior to loading the samples in the microscope they were coated with 20 nm gold to reduce charging effects. After the aerosol’s filtration test, a random area of the masks with no dye detection and the region with the major passage of dye were selected for analysis on all layers detached.

## Results

### Qualitative and quantitative analysis of masks filtering

All groups presented some level of aerosols passage (Table 1). Fava Masks presented the lowest number of masks with dye staining (55%), whereas MaxClean, Descarpack and Elite Professional groups had the highest levels of aerosols passage (88.8%). Regarding quantitative analysis, significant differences between areas of aerosols’ passage through masks were observed (*p* < 0.05). Elite Professional and Ortho Pauher masks showed the best results between evaluated groups. In contrast, Descarpack and MaxClean showed the larger areas of aerosol passage with no difference between them.

**Table 1:** Percentage (frequency) of surgical masks detected with aerosol passage and area of aerosol passage through masks in all groups

Groups	Qualitative analysis - % (*N*)	Quantitative analysis - Area (cm^2^)
MaxClean	88.88% (8)	9.05 ^D^
Descarpack	88.88% (8)	3.16 ^CD^
Elite Professional	88.88% (8)	0.15 ^A^
Sol-Millennum	77.77% (7)	2.20 ^ABC^
Neve	66.66% (6)	1.09 ^ABC^
Ortho Pauher	66.66% (6)	0.12 ^A^
Phitta mask	66.66% (6)	0.4 ^AB^
Fava Mask	55.55% (5)	0.71 ^AB^

Different letters^ABCD^ indicate statistically significant values between groups in the quantitative analysis (Dunn test, *p* < 0.05)

### Microscopy analysis of masks

**Fig. 4:**
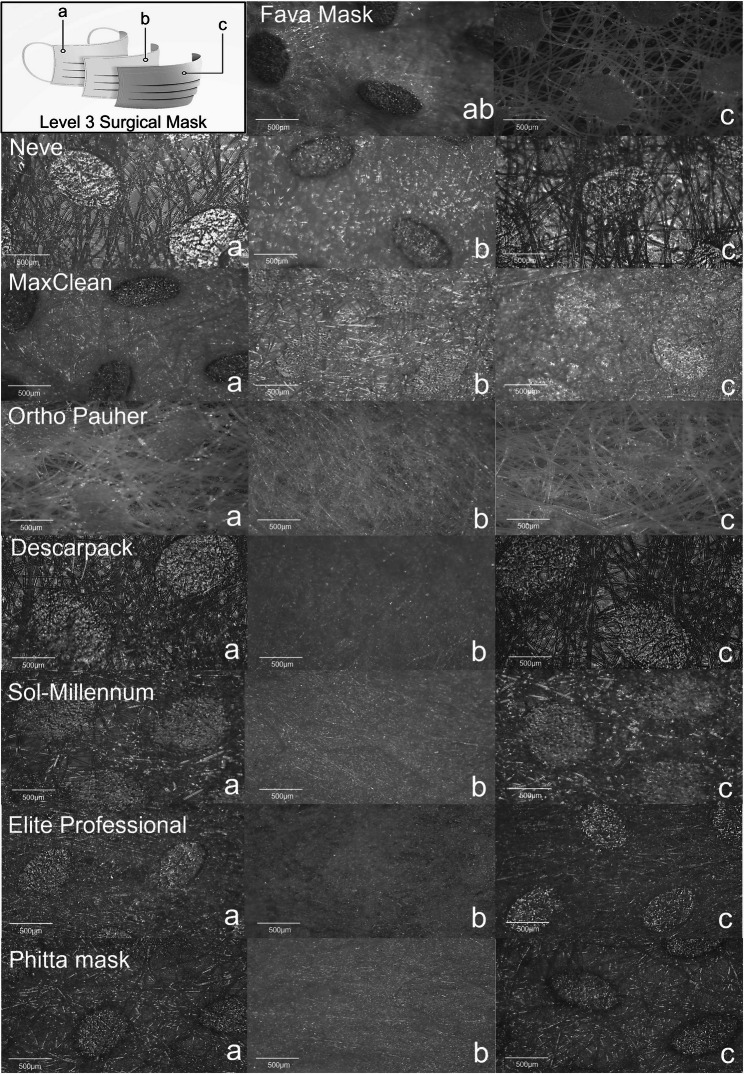
Stereoscopic representative micrographs of (**a**) inner, (**b**) middle and (**c**) outer layers of the groups of masks evaluated (bar scale: 500 µm)

**Fig. 5:**
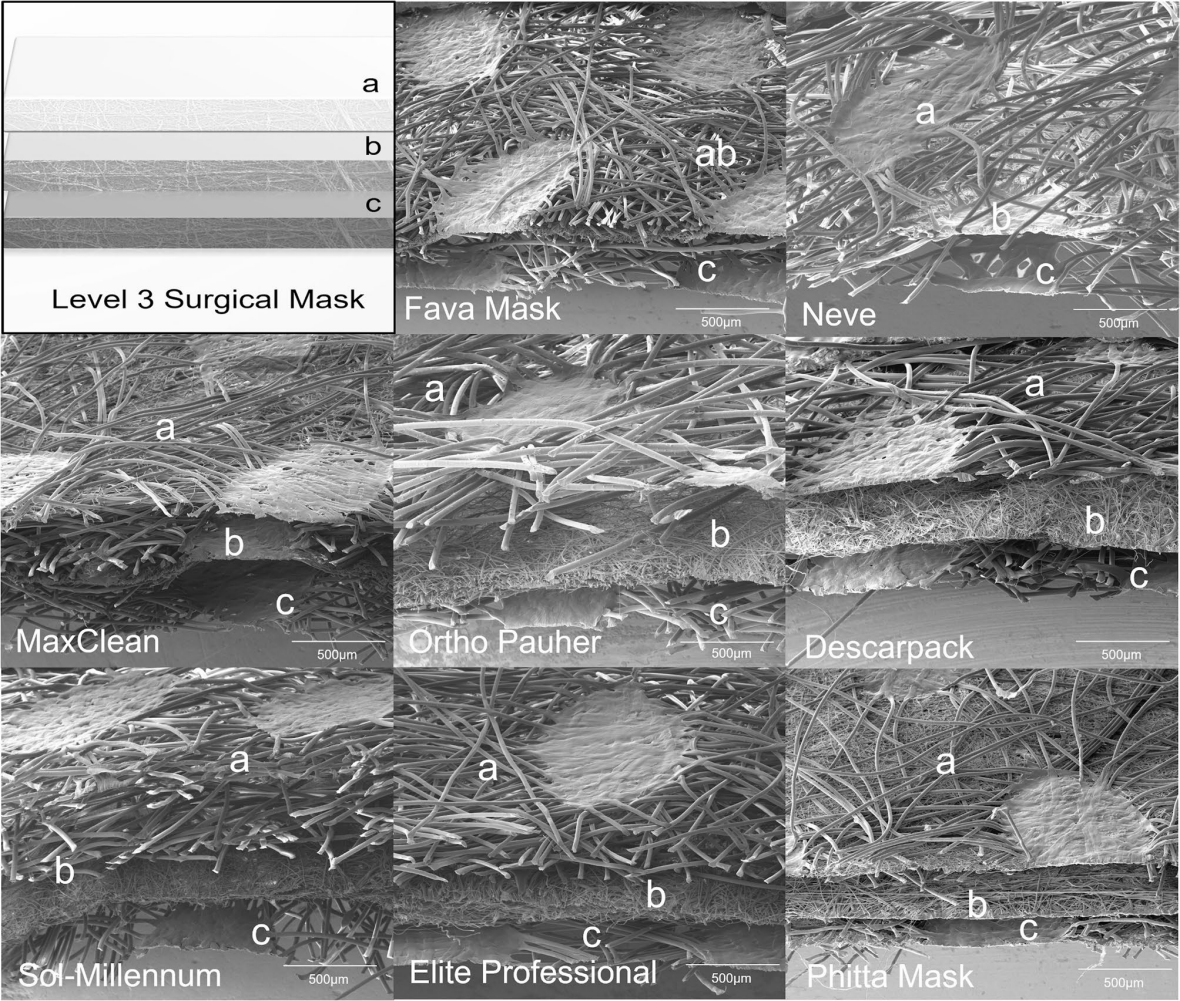
Cross section images of the groups of masks evaluated by scanning electron microscopy showing (**a**) inner, (**b**) middle and (**c**) outer layers (bar scale: 500 µm)

**Fig. 6:**
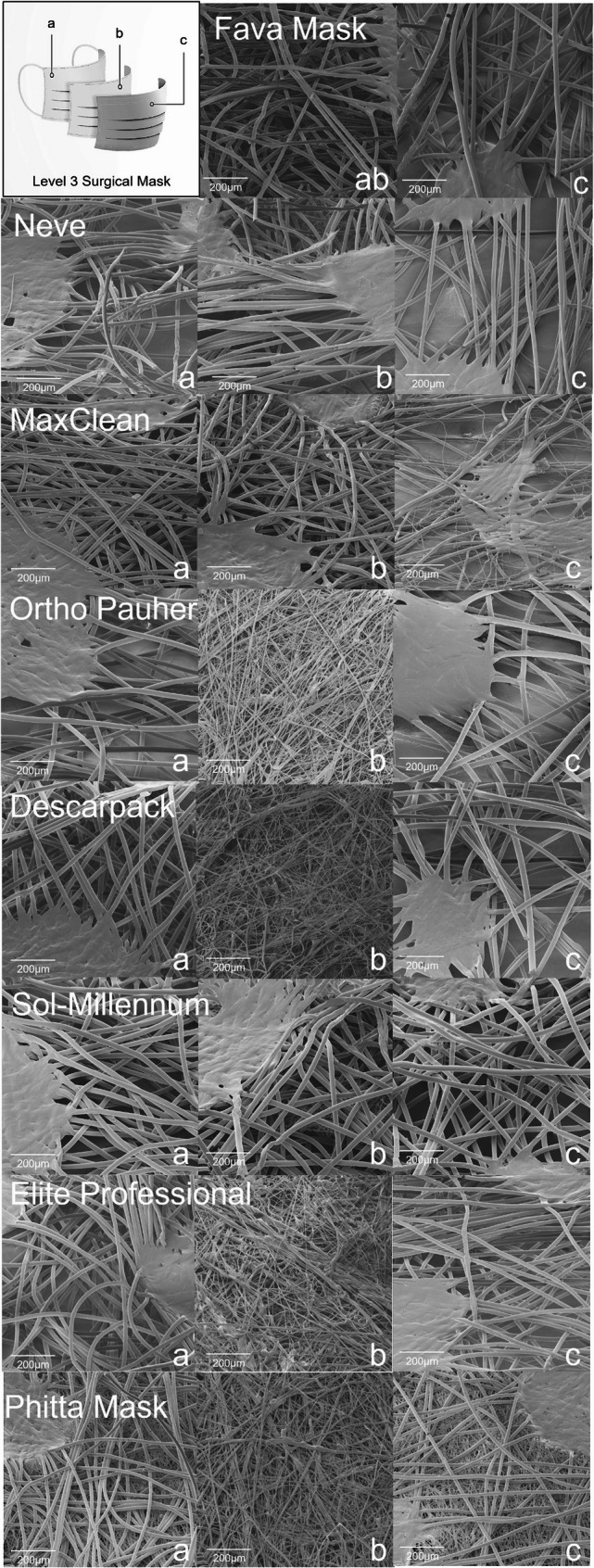
Representative images by scanning helium ion microscope of (**a**) inner, (**b**) middle and (**c**) outer layers of the groups of masks evaluated (bar scale: 200 µm).

**Fig. 7:**
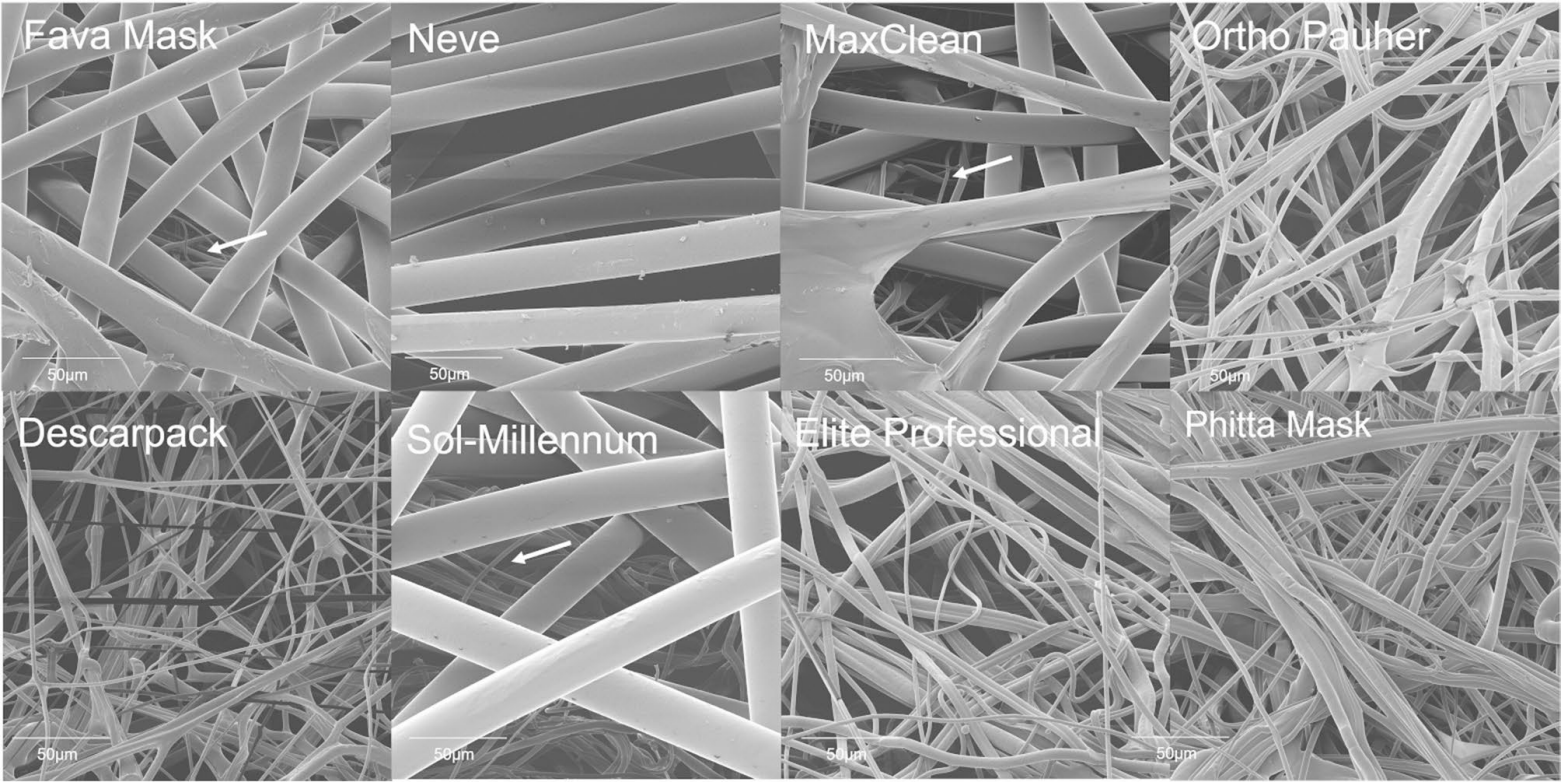
Middle/filtering layer of masks in high magnification by scanning helium ion microscope (bar scale: 50 µm). * : overlapped layer with thinner fibers

Representative morphological images of groups are presented in Figs. 4, 5, 6 and 7. No difference was observed among stained and non-stained areas of the masks. Most masks evaluated in this study were SM (inner and outer layers = spunbond; middle layer = meltblown) materials.

- Fava Mask: Although Fava Mask is an SM material, only two layers were observed. The middle layer was attached with the inner layer, producing an inner-middle layer sheet denser than the outer layer, showing numerous fibers associated in multiple levels.
- Neve: All three layers are similar with spunbond processing aspect, but the middle layer seems denser in comparison to the inner and outer layers. Higher magnifications of analysis proved the overlapped presence of fibers with smaller dimensions in middle layer. Therefore, middle layer showed patterns of both spunbond and meltblown process.
- MaxClean: All three layers are similar with spunbond processing aspect, but the inner layer seems denser in comparison to the middle and outer layers. Higher magnifications of analysis proved the overlapped presence of fibers with smaller dimensions in middle layer. Therefore, middle layer shows patterns of both spunbond and meltblown process.
- Ortho Pauher: SM material. In comparison to other groups, the middle layer seems thinner with a lower number of fibers.
- Descarpack and Elite Professional: SMs materials. Different proportions of fibers can be observed between groups, underscoring differences in the development process of masks.
- Sol-Millennum: Although optical microscopy of Sol-Millennum shows patterns of a SM material, SEM revealed presence of spunbond characteristics in the middle layer. Higher magnifications of analysis proved the overlapped presence of fibers with smaller dimensions in middle layer. Therefore, middle layer showed patterns of both spunbond and meltblown process.
- Phitta mask: SM material. Higher magnifications of layers analysis proved the overlapped presence of fibers with smaller dimensions in inner and outer layers. Therefore, inner and outer layers show patterns of both spunbond and meltblown process.

## Discussion

The inability of researchers to mimic real environmental parameters of aerosols production and filtration via laboratory experiments, field tests, and in vitro/in vivo investigations is one of the main current challenges to fully understand the efficiency of masks [27]. Moreover, the traditional foundation of airborne transmission neglects the relevance of aerosols inhaled at close range to an infected person during dental treatment, where exposure is more likely [28, 29]. 

A series of studies, mainly up to early 1990 s, attempted to evaluate the protection ability of masks during dental care [6]. Most of the previous studies used culture-based methods to assess contamination distances from the dentist point-of-view [10, 30]. In 2005, the relative filtering efficacy of masks were analyzed in a dental clinical setting by spraying bicarbonate particulate against a porcelain surface followed by the calculation of the dry residue weight [6]. More recently, the filtering efficiency of masks against composite dust produced with high-speed handpiece was assessed in an individual personal inhalable aerosol sampler in a set-up with no water spray [31]. However, to the best of the authors knowledge, this is the first study to assess aerosols’ passage through masks in a modern dental office setting using high-speed equipment with the patient seated in a reclining position and the dentist presenting human breathing.

This simulation model used in this study presents some methodological aspects that should be discussed. As aerosols are commonly loaded with microorganisms [29], the use of a red dye on the dental equipment water allowed the visualization of aerosols passage and thus the contamination risk for dentists [31]. Red dyes have variable particle sizes, with FD&C Red No. 40 having an average particle size of 100 micrometers [32], much larger than some species of bacteria and viruses, showing that this degree of contamination could be even greater if microorganisms had been evaluated. Additionally, this method, using red dye, was recently used for Barboza et al. in 2020, to evaluate the passage of aerosols and droplets generated by handpieces during dental procedures through different non-woven weights [26]. 

To our knowledge, for the first time, an in vitro system simulated human conditions such as breathing and the use of a 3D printed human face, conditions that can affect the mask’s seal and the passage of aerosols. Although these are great advances, it is worth noting that there are other non-simulated conditions as professional movements during care, breathing humidity and body heat.

Anthropometric analysis of dental professionals aimed a more reliable simulation of dental clinical setting and the standardization testing between groups. The recommended ergonomic posture for dentists [33] was applied in this study but adopting inadequate positions during dental care may lead to higher levels of aerosol passage through masks [34].

The proposed model allowed the standardization of aerosols exposure to the dentist’s face, including the type of aerosol, particle size, speed and amount. However, it is known that the distribution of aerosols is extremely variable and may be influenced by many factors such as the type of dental procedure and the position of the tooth in the mouth [3]. In the present study, we chose to evaluate only one dental procedure, which has previously been identified as the one that generates the most aerosols [22, 26]. However, future studies could evaluate other procedures, as well as the transmission of microorganisms.

In the present research it was decided not to use face shield combined with SM, since according to Remington et al. 2022 it did not appear to offer any protection from aerosolized particles [35]. Etchatz et al. [36] observed that professionals began to abandon the use of face shields due difficulties in viewing, blurring and annoying. Although the use of high suction can influence the results [35], not all dentists have access to high and external suction.

In this study, masks showed an alarming 55% minimum level of aerosols passage indicating that the dental operator is exposed to considerable potential respiratory hazard. Therefore, no level 3 SM was considered adequate. This finding is in accordance with recent studies that also observed a low filtering efficiency of SM [14, 37]. Even when properly sealed, medical masks cannot completely block the passage of aerosols [38]. It is recommended that masks present the minimum level of droplets filtering efficiency of 95% to ensure protection [39]. This falls within the reasoning that improvements are required for standard precautions to avoid risk of cross-infections during dental care.

The performance of masks was different among qualitative and quantitative analyses. Elite Professional presented a high percentage of masks showing aerosol passage (88%), but a small area of aerosol passage (0.15 cm^2^). Therefore, Elite Professional proved intermediary results of filtering efficacy. MaxClean and Descarpack groups showed the worst results with high levels of aerosol passage (88.8%) and large areas of aerosol pigmentation (9.05 cm^2^ and 3.16 cm^2^, respectively). Neve, Ortho Pauher, and Phitta mask showed intermediate results with 66% of masks with aerosol passage and small areas of aerosol passage (1.09 cm^2^, 0.12 cm^2^ and 0.4 cm^2^, respectively). Moreover, Fava Mask was considered the best group with the lowest level of aerosol passage (55%) and a small pigmentation area (0.71 cm^2^). The differences in groups can be explained by masks composition and processing technologies used.

SM are typically composed of layers of nonwoven fabrics that are produced from thermoplastic polymers [28]. The level 3 SM contain 3 layers that ideally present the following characteristics: (a) an inner layer – in contact with the wearer face – based on soft fibers of a hydrophilic material, (b) a middle hydrophobic layer that consists of the main filter of bacteria and viruses, and (c) an outer layer exposed to the environment with hydrophobic properties [39]. 

Several processes can be used for the development of nonwoven polymeric fabrics but the most common are the spunbond and the meltblown processes, which comparatively present clear differences in morphology and properties [28]. The spunbonded fabrics consist of coarser filaments with greater tensile strength and smaller pressure drop that are developed by a two-step process [40]. The meltblown process is a simple and versatile one-step method that directly transforms polymeric raw materials into membranes of nonwovens fiber [41]. Most masks evaluated in this study were SMS materials as observed in microscopy. That was in accordance with what was reported by the manufactures. All masks showed the inner and outer layers produced by spunbond technique. Nevertheless, the middle layer of masks presented either spunbond or meltblown fabrics. It was expected that only meltblown process would be found in the main filtering layer because of the production of fibers that allows reduced submicrometer dimensions (1–2 μm) [41], which permits a higher surface area and improved filtering performance [28]. 

The Fava Mask, Neve, MaxClean and Sol-Millennum showed spunbond characteristics in the middle sheet of masks, but only MaxClean showed unsatisfactory results in aerosols passage. The outer layer of MaxClean presents different patterns from the other groups because of a thinner sheet and fibers of different dimensions that are apparently more fragile. Similarly to MaxClean, Descarpack also showed inferior results despite presenting a SMS composition. This may result from the properties of polymers selected for producing nonwoven fabrics since this composition reflects on shape, degree of absorption, and pore size of masks. Furthermore, although Fava Mask, Neve, and Sol-Millennum have a spunbond feature in middle layer, they also showed an overlapped layer with thinner fibers constituting a dense filtering layer. Therefore, it could be assumed that middle layer of Fava Mask, Neve, and Sol-Millennum present two sheets with both spunbond and meltblown process each. These masks could also have been submitted to a different technique in only one sheet. Fava Mask manufacturer claimed by personnel communication that this mask is a SMS material produced through a process that adheres the inner layer to the middle layer. The referred assumption could be associated to the comparatively better results of filtering efficiency of this mask.

In this investigation, samples were characterized topographically by different microscopic techniques. These analyses revealed that inner and outer layers of SM were similar, but middle layer varied. Material chemistry as fiber polymer types and electrostatic charge and other analyses can be consider in future works.

## Conclusion

The inner and outer layers of masks are topographically similar, different to the middle layer. All level 3 SM presented high levels of aerosols passage when a simulation model of dental care with human breathing system was used.

## Data Availability

All data generated or analyzed during this study are included in this published article [and its supplementary information files].

## Abbreviations

ABS: Acrylonitrile butadiene styrene
ASTM: American society for testing & materials
COVID-19: Coronavirus
FDM/FFF: Fused deposition modeling/fused filament fabrication
HIM: Helium ion microscopy
RPM: Respirations per minute
SEM: Scanning electron microscope
SM: Surgical masks
